# Supplementary material for: Maresin-1 promotes neuroprotection and prevents disease progression in experimental models of multiple sclerosis through metabolic reprogramming and shaping innate and adaptive disease-associated cell types
Source: bioRxiv. 2024 Jun 19:2023.09.25.559216. Originally published 2023 Sep 26. Preprint. [Version 2] doi: 10.1101/2023.09.25.559216 (PMC10557612; doi:10.1101/2023.09.25.559216)
Supplement: 1 [file NIHPP2023.09.25.559216v2-supplement-1.pdf]

Supplementary information

Maresin-1 promotes neuroprotection and prevents disease progression in experimental models of multiple sclerosis through metabolic reprogramming and shaping innate and adaptive disease-associated cell types

Insha Zahoor[1*], Mohammad Nematullah[1*], Mohammad Ejaz Ahmed[1], Mena Fatma[1], Sajad Mir[1], Kamesh Ayasolla[1], Mirela Cerghet[1], Suresh Palaniyandi[2,3], Veronica Ceci[4,5], Giulia Carrera[5], Fabio Buttari[6,7], Diego Centonze[6,7], Yang Mao-Draayer[8], Ramandeep Rattan[9], Valerio Chiurchiù[4,5], Shailendra Giri[1#]

Supp Figure 1

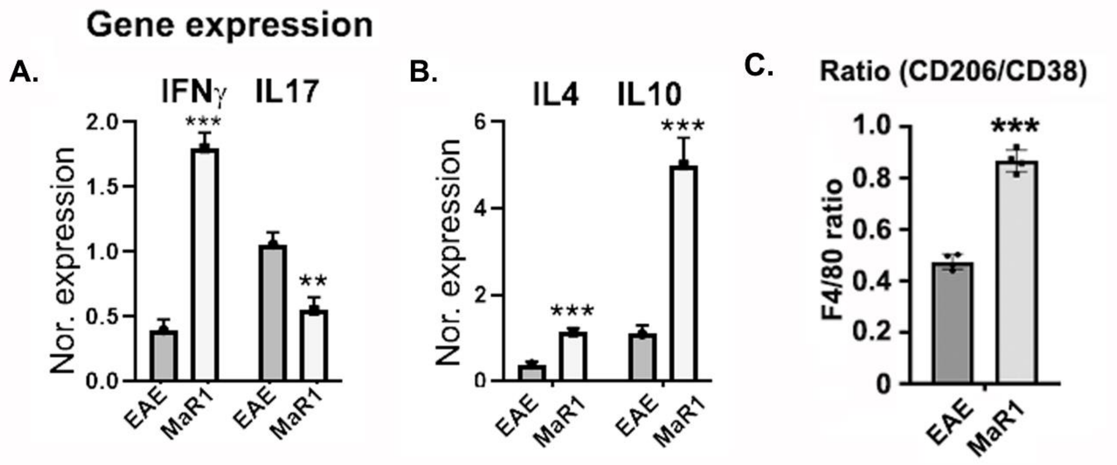

Supplementary data

Supp Fig. 1: MaR1 modulated the expression of pro- and anti-inflammatory cytokines in spleen/LN cells and altered the phenotype of macrophages in the CNS. A-B. RNA was isolated from spleen/LN cells from mice with untreated EAE and MaR1 after 24 h of antigen stimulation, after which the expression of IFNγ, IL17a, IL4 and IL10 was examined (n=3). **C.** CNS tissues (brain and spinal cord together) were processed, and CD206+ and CD38+ F4/80+ cells in the CNS of treated and untreated RR-EAE (n=4) were examined. The ratio of CD206/CD38+ macrophages was plotted to determine the macrophage phenotype (n=4). ***, P<0.01.

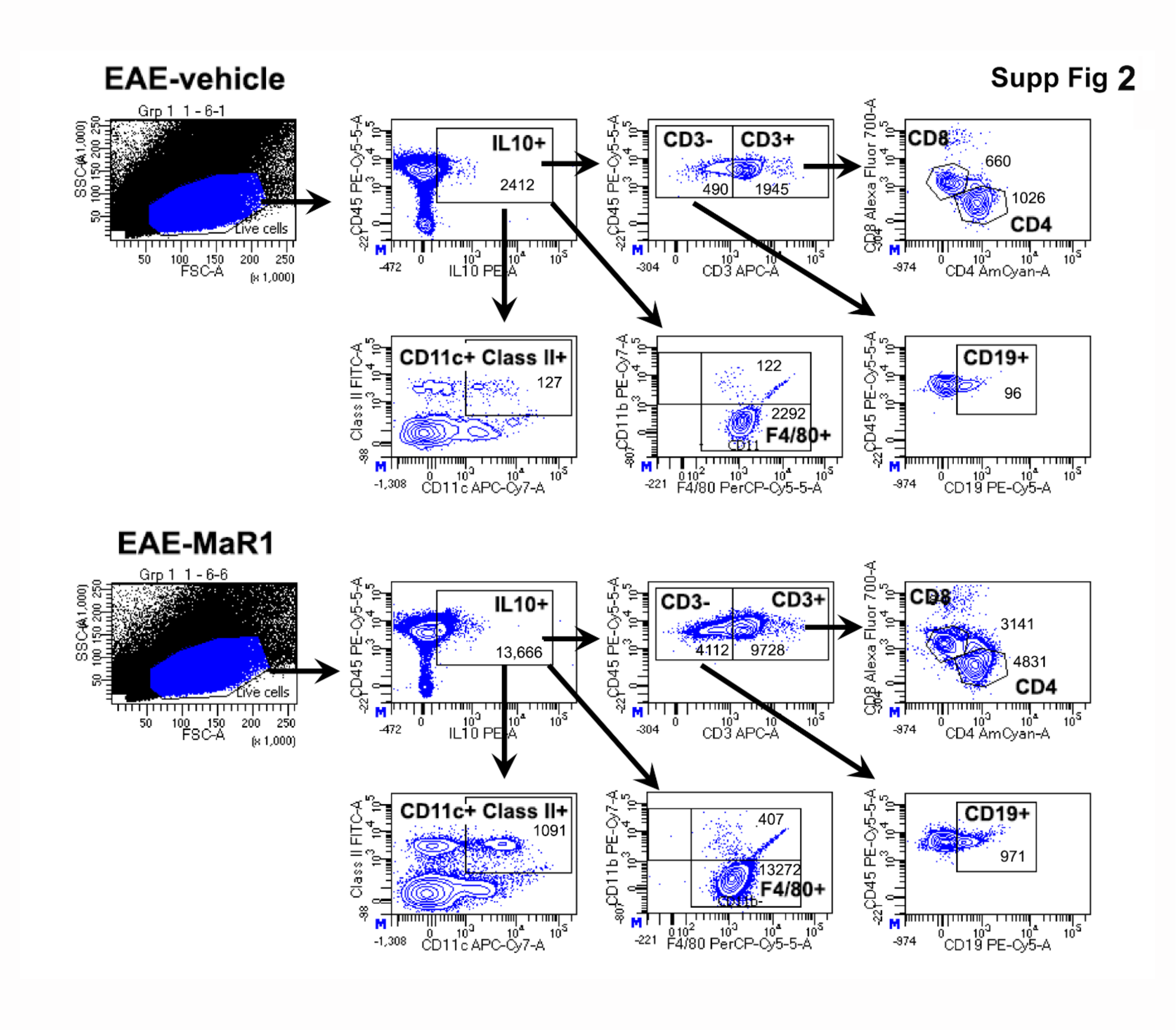

Supp Figure 2: Gating strategy for examining IL10-expressing immune cells. Using a Percoll density gradient, brain and spinal cord single-cell suspensions from EAE and MaR1-treated EAE mice were generated and subjected to flow cytometry analysis. Before identifying IL10-expressing lymphoid cells, CD3+ populations were gated from IL10+ populations. CD4+ and CD8+ T cells were identified in CD3+ populations, while B cells were identified in CD3- populations. To identify IL10-expressing myeloid populations, CD11c+ClassII+ populations were gated for inflammatory dendritic cells, whereas CD11b+ F4/80+ populations were gated for macrophages from IL10+ populations.

Supp Fig 3

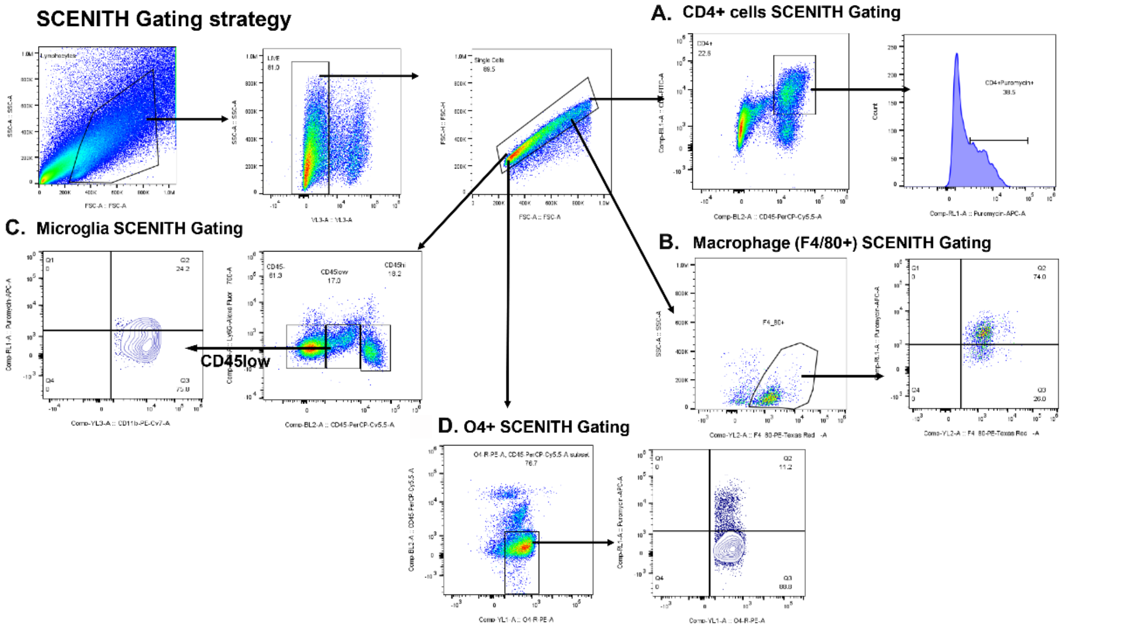

Supp Fig 3: Gating a strategy for immunophenotyping using SCENITH. The brain and spinal cord single-cell suspensions were stained for lymphoid and myeloid markers, as described in the methods section. To eliminate doublets and dead cells, the Live_Dead-ve population was gated, and the FSC-A vs FSC-H data were plotted from negative populations, identifying these populations as live cells, which were used in all subsequent analyses. CD4+ T-cell populations were identified by double gating of CD45+CD4+ cells, and puromycin-positive cells were gated from this population. Microglial populations were gated based on CD45lowCD11b+ and puromycin double-positive cells. To detect puromycin+ macrophage populations, CD45+F4/80+ cells were gated for total macrophages, followed by puromycin-positive populations.

Supp Figure 4

***In Vitro* microglia efferocytosis Gating**

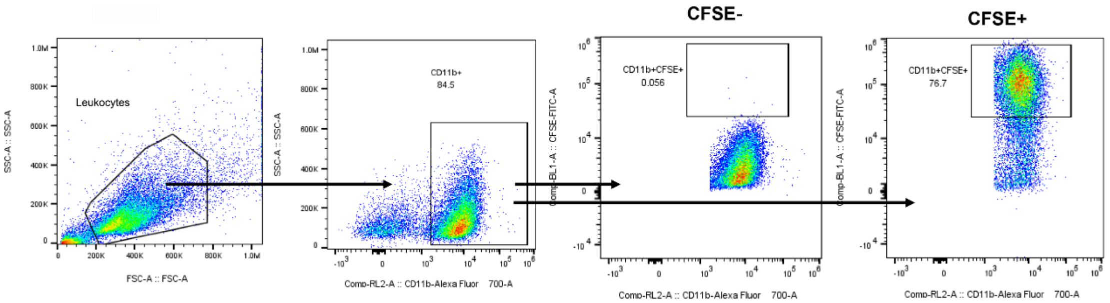

Supp Fig 4: Gating strategy for e*x vivo* efferocytosis analysis. A pseudocolor map was used to quantify total efferocytosis in macrophages and microglia from mice subjected to EAE and treated with MaR1. Double-positive gating of CD11b+CFSE+ populations revealed apoptotic cell engulfment by macrophages and microglia, whereas CD11b+CFSE- populations were identified as nonefferocytotic macrophages and microglia.
